# Early Digital Engagement Among Younger Children and the Transformation of Parenting in the Digital Age From an mHealth Perspective: Scoping Review

**DOI:** 10.2196/60355

**Published:** 2025-07-03

**Authors:** Nafisa Anjum, Md Mehedi Hasan, Nursat Jahan, Sheikh Iqbal Ahamed, Allison Garefino, Nazmus Sakib

**Affiliations:** 1 College of Computing and Software Engineering, Kennesaw State University, 1100 South Marietta Parkway, Kennesaw, GA, 30060, United States, 1 4147975981; 2 Department of Computer Science, Marquette University, Milwaukee, WI, United States; 3 Department of Psychology, Kennesaw State University, Kennesaw, GA, United States

**Keywords:** infants and toddlers, screen time, cognitive development, parental ignorance, intervention strategies, mHealth, digital addiction, children, developmental challenge, parents, engagement, decision-making

## Abstract

**Background:**

Evidence identifies that excessive screen time consumption during the crucial stage of life (0‐3 years) significantly affects children’s holistic development over time. In today’s intricate socioeconomic setting, parents, especially working parents, face challenges in constantly supervising their children’s activities, often turning to digital devices as a suitable substitute to keep them occupied. To address these issues, a mobile health (mHealth) app can emerge as a feasible solution to help parents manage digital habits for their infants while minimizing the harmful effects.

**Objective:**

The aim of this scoping review from an mHealth viewpoint is to raise awareness among parents about the detrimental effects of unwarranted screen exposure in children younger than 3 years and recommend effective strategies for redirecting them to alternative developmental activities, promoting balanced digital engagement for their infants and toddlers within their domestic landscape.

**Methods:**

A systematic search of academic databases, including Google Scholar, PubMed, IEEE Xplore, and Elsevier, was conducted. To discover existing child screen monitoring apps, searches were conducted in the Google Play Store and Apple App Store through specific keywords across regional marketplaces. The PRISMA (Preferred Reporting Items for Systematic Reviews and Meta-Analyses) guidelines were followed to organize the literature search process. Data collected from the studies were organized into a predeveloped Excel spreadsheet to facilitate analysis. Synthesized data were scrutinized to detect patterns, variances, and reasonable recommendations.

**Results:**

While parents acknowledge the negative impacts of young children’s excessive screen time, their dependence on digital devices survives due to today’s modern lifestyle commands. In total, parents’ insights were clustered into 9 separate categories, highlighting that parents often believe smart devices are beneficial for their children. A total of 6 intervention approaches for parents and 3 for pediatricians were summarized. A significant finding was parents’ unawareness of the association between their own screen time and their toddlers’ interactions with screen media. Additionally, parents also perceived existing intervention strategies positively and acknowledged them as helpful solutions. However, they also recognized that inadequate tools and insufficient time for execution caused the gap in these approaches.

**Conclusions:**

The findings of this study underline the need for an empathetic tool to help parents manage their children’s screen time efficiently. The development of a holistic mHealth app is presented that considers awareness, practical guidance, and personalized interventions to balance children’s digital device use. The proposed solution could incorporate four essential features: (1) screen time tracking and monitoring, (2) a reservoir for parental training and guidelines, (3) an alternative activity advocator, and finally (4) an interactive artificial intelligence assistant. This study provides valuable insights into improving obedience to healthy screen use and fostering a digital ecosystem where technology itself functions as an advocate of child progress, instead of an obligation.

## Introduction

The escalating inclination towards extreme screen time use among children has become a universal problem recently, with evidence pointing to imposing severe impacts on children’s early childhood development, for instance, lower academic achievements, increased anxiety, difficulties in language acquisition, and other developmental setbacks [1-4]. The American Academy of Pediatrics (AAP), a leading authority dedicated to safeguarding children’s welfare, restricts any form of screen media exposure for children younger than 2 years [5]. Nevertheless, studies have uncovered that almost 68% of children younger than this age bracket often surpass this threshold, spending around 2.05 hours of screen time each day, which is overwhelming [6,7]. In addition, watching television, one of the most conventional modes of screen media, for more than 2 hours per day was observed to be 83% in the United States, 78% in Australia, and 82% in Canada among toddlers aged 2 to 5 years, contributing to a sedentary lifestyle within approximately 34% to 94% of children, which are equally alarming [7,8].

The complex socioeconomic landscape further intensifies this issue, as modern parents face an array of obstructions in constantly monitoring their children’s activities. Parents play a central role in influencing their young children’s screen time habits, as their screen observation levels and behavior are greatly related to those of their children. Their positive attitudes toward screen time can unconsciously increase screen exposure to their young children, leading to behavioral imitation and delays in developmental outcomes [9]. Contrariwise, research indicates that reduced parental screen time is associated with reduced screen exposure in children younger than 3 years, increasing parent-child interactions that are crucial for early cognitive and emotional development [10]. With the limited scope of supervision, screen devices have emerged as a convenient child supervision tool, performing as a companion for struggling parents, leading to approximately 70% of parents ignoring the AAP recommendations, unintentionally contributing to the challenge of screen time management [11,12]. These observations highlight a disconnect between existing guidelines for limiting screen time in infants and the practical challenges parents encounter in limiting digital device use, which implies the need for a detailed reassessment of prevailing strategies to promote a balanced lifestyle that is practical for parents and protective of children’s optimal growth and developmental needs.

**Figure 1.**
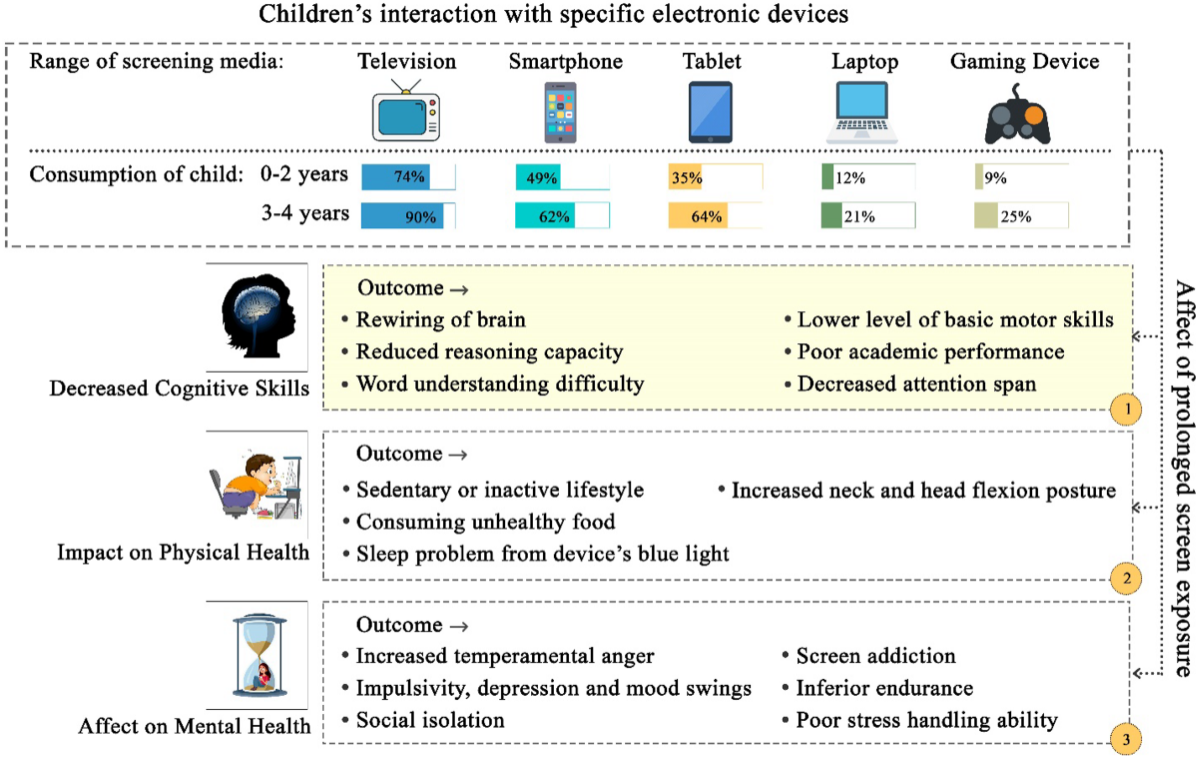
Influence of excessive screen time on early childhood developmental spheres.

The foundations for effective intellectual and analytical skill advancement are embedded in early childhood, which offspring usually adopt and develop within the initial 3 years of life [13]. However, throughout this critical stage, infants and younger toddlers are also enormously vulnerable to the harmful consequences triggered by long-lasting screen exposure [14]. Evidence implies that consuming a higher rate of screen time negatively impacts children’s cognitive growth with a downstream influence on their physical, academic, and psychological outcomes, as illustrated in Figure 1 [15-17]. A psychological study from Korea involving kids between 24 and 30 months of age displayed a proportionate relationship between prolonged screen time and delays in language development and challenge-solving skills [6]. Similarly, higher television exposure at only 29 months of age was correlated with minimum vocabulary acquisition levels at a later age [18]. On the contrary, research has exhibited that watching high-quality programs under parental supervision has advantages, including attention span and vocabulary improvements, compared with children who do not watch such content at all. Such quality content often uses tactics including findable object labeling, proper formatting, the interaction between the child and characters, and a structured program outline, which can help in their developmental benefits [19]. Nonetheless, many of these affirmative statements targeting children younger than 2 years remain unproven, as stated in the 1999 AAP policy announcements [2,20,21]. These opposing outcomes infer a huge gap in our understanding and the need for an adapted rather than a one-size-fits-all answer.

Previous studies regarding the potential consequences of screen time have largely targeted the age range of children between 5 and 7 years; nonetheless, limited attention has been given to children between 0 and 36 months. This study argues that instead of completely discarding the abundant presence of screen devices, it is crucial to adopt a parent-centric, rational strategy that aligns with today’s contemporary lifestyles. This paper proposes that the development of a mobile health (mHealth) app can enable parents to manage their children’s screen time and maintain a balance between technology consumption and mindful parenting. This study aims to acknowledge the research gaps, emphasize the existing blind spots, and offer appropriate solutions. To accomplish this, the paper addresses the following fundamental research questions:

RQ1: How does the absence of knowledge regarding the prospective threats of excessive screen use influence parental decision-making procedures?

RQ2: What alternative approaches have been identified that parents can successfully use to divert their children from unnecessary screen time while reinforcing their intellectual, physical, and emotional expertise in a home-based surrounding?

RQ3: How can these alternate strategies be seamlessly integrated into a holistic approach that mitigates the adverse effects of excessive screen exposure on young children’s overall development?

We start by representing a systematic approach to reviewing existing and appropriate literature in the designated field, analyzing their insights, and revealing critical knowledge gaps in parental understanding regarding screen time management. Building on these insights, we present some actionable suggestions and explore the capability of developing a mHealth app as a rational tool for parents to help them create a better and healthier digital balance for their children younger than 3 years.

## Methods

### Overview

Through a structured methodology, broad topics can be investigated and blind spots in the existing literature can be identified [22]. We conducted this research using the methodological framework for scoping reviews proposed by Arksey and O’Malley [23], ensuring a meticulous and transparent approach. The methodological framework of this research is designed to provide a comprehensive understanding of the central research questions by identifying primary studies through different sources. Our framework is divided into 3 distinct phases, covering a thorough scoping criteria, a systematic literature search, and a data synthesis process, which are rigorously carried out to ensure alignment with our research objectives.

### Scoping Criteria

In phase 1, we identified studies inspecting the reasoning behind parents’ dependence on smart devices, their impact on children’s early development, and the viability of alternative intervention activities. The study, furthermore, accounts for parents’ misunderstandings regarding the positive effects of screen media consumption on their children’s health development, alongside how their own digital habits influence their children’s media use patterns. In this research, all contributory studies related to the critical research questions offering insights into the behavioral and developmental implications of excessive screen use received preliminary approval. This phase was performed to encourage parents’ early involvement in using effective solutions, preceding a more thorough investigation of the problem.

### Systematic Literature Search

In phase 2, we used a systematic literature search to identify studies that complemented the work done in phase 1 and focused on suggesting, describing, evaluating, and implementing novel insights into mitigating screen dependency on children younger than 3 years. The literature review contained journal papers, peer-reviewed articles, and official reports obtained from academic databases such as JMIR, Google Scholar, IEEE Xplore, Elsevier, and PubMed. Advanced search features, such as MeSH terms in PubMed, along with targeted searches including Pediatrics and Parenting, mHealth and uHealth within JMIR were used to recognize the role of child- and parent-centered approaches using mobile apps, information technologies, engineering solutions, and expand the scope of infants and toddlers’ health outcomes. We then formulated a series of specified keywords stemming from the research questions to group papers across the abovementioned databases, as detailed in Table 1. Additionally, to gather information on publicly available parental child monitoring mobile apps for smartphones, we conducted searches of the Google Play Store and Apple App Store using keywords like “Family,” “Child,” and “Safety” across regional marketplaces.

**Table 1.** Search strategy.

Search approach	Field	Context	Motivation
Primary search term	Screen use and child development	Early childhood screen use	Parental monitoring solutions
Synonyms	“infants,” “young toddlers,” and “early childhood”	“cognitive growth,” “psychological development,” “sedentary lifestyle,” “screen time,” “factors and dominance of screen viewing,” “risks of extreme screen media,” and “alternate solutions”	“mHealth,” “software system,” “software application,” and “safety”

Publications were eligible for inclusion if they were written in English, issued in interdisciplinary conference proceedings, satisfactory journals, or as official reports formed by a government constitution. Broad eligibility criteria were used to maximize the possibility of finding appropriate information, such as the studies that demonstrated expertise in child health science and child psychology, explored alternative strategies to reduce screen dependency such as outdoor activity or mHealth apps, and involved in a discussion related to the detection of effects, connections, and solutions for children younger than 36 months. Researchers applied exclusion criteria to ensure consistency in screening and to maximize the possibility of excluding unsatisfactory literature. The exclusion process involved 2 stages of screening, an initial removal of duplicate and irrelevant articles based on titles and abstracts followed by an exclusion of studies that were not peer-reviewed, published in gray literature, and unrelated especially to child cognitive or behavioral growth, resulting in the elimination of approximately 48% publications of the primary pool.

### Data Synthesis Procedure

The data synthesis was performed to categorize the selected papers for evidence synthesis and address the research questions briefly. Data collected from the studies included were organized into a predeveloped Excel spreadsheet to facilitate analysis. The extracted data encompassed bibliographical data (eg, author, title, year of publication, DOI), study characteristics (eg, type of study and sample demographics), contextual information (eg, screen media type, parental decision-making factors, perceived developmental impacts), and evaluation criteria (eg, parental behaviors, child performance, intervention strategies, and outcomes such as cognitive, behavioral, physical, and social development). Classification formats were developed to organize the efficacy of interventions (eg, screen time reduction, improved parental engagement, and coviewing) and their relevance to developmental outcomes. Data charting involved systematically populating the metadata from each study into predefined categories. Key findings were reviewed to ensure accuracy. The synthesized data underlined crucial insights: parental fallacies regarding screen time, gaps in existing policies, and the necessity for accessible, technology-motivated solutions. This phase highlighted the similarities and discrepancies across selected literature, offering recommendations that address the research questions and confirming that this study’s conclusions achieve optimal reliability and scalability in diverse childcare settings.

## Results

### Overview

**Figure 2.**
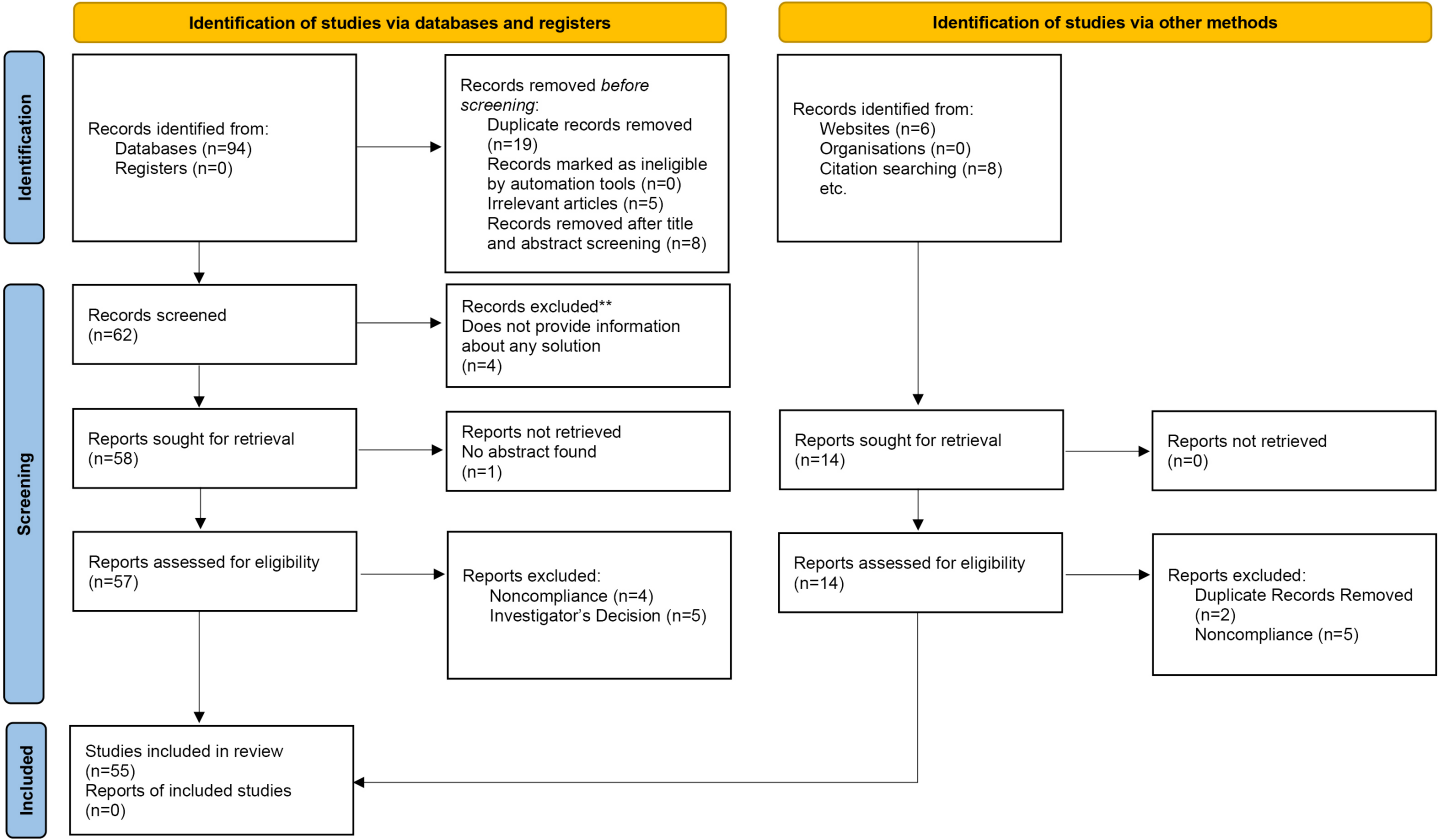
PRISMA (Preferred Reporting Items for Systematic Reviews and Meta-Analyses) flowchart displaying systematic literature search.

This scoping review tries to answer research questions presenting the significance of parental attitudes, perspectives, and practices related to screen use that influence the screen time pattern of their infants and toddlers. This study reveals that parents’ lack of awareness regarding their own habits of screen use directly affects their toddlers’ relationship with smart devices, impeding their healthy development. Hence, these outcomes highlight the necessity to educate parents on the implications of screen use to promote well-informed decision-making that can draw the odds out of screen device use. The results also highlight the importance of balanced use of screen time by children due to the modern parenting environment. The study selection process is illustrated in the PRISMA (Preferred Reporting Items for Systematic Reviews and Meta-Analyses) flowchart, as shown in Figure 2, which outlines the records identified, screened, and included in the final analysis.

### Significance of Consciousness in Parental Decision-Making (RQ1)

The findings from reviewed studies reveal the outcomes related to early childhood screen exposure, highlighting that the majority of parents remain unaware of how their own screen use directly influences the screen time behavior adopted by their infants and young toddlers [11,24]. As the principal guardians and decision makers, parents uphold a momentous role in shaping their children’s early interaction with smart devices, who are tremendously susceptible to the potential consequences of excessive screen exposure during this critical development stage [13]. Thus, to construct more rational and informed guidelines regarding screen time limitations, it is essential to further investigate parental decision-making procedures, incorporating their values and theories in today’s modern settings. In Table 2, we categorized parents’ perceptions towards their positive beliefs in smart devices for their children. The results of children’s screen viewing depend heavily on wrong impressions of their parents’ decisions regarding the age inappropriateness of content, exposure to background media, allowing screens during mealtimes, and so forth. For example, studies have identified that regularly exposing a toddler to noise made from television or other digital content playing in the background while a child is engaged in another activity can disrupt their focus, reduce verbal interactions, and delay vocabulary growth [15,20,25,26]. Similarly, dependence on any mode of screen device during sleep time often results in reduced parent-child bonds and a disrupted sleep pattern.

**Table 2.** Observations of parents as opposed to their actual impact.

Parents observations	Decision-making factors	Effects on cognitive development	Additional impacts	Reference
Protected from externa**l** world	Parents from unsafe neighborhoods prefer providing screen time to keep their toddlers indoors.	Higher screen dependence at school age Less attention span	Higher risk of obesity Aggressive behavior	[2,27]
Screen media as an educational tool	Confidence that programs that target younger toddlers are educational and enhance child development.	Decreased child vocalizations. No considerable linguistic abilities Reduced school-connectedness	Inverse impact on self-esteem.	[2,6,15,28]
Background media use	Children exposed to adult contents (termed as background noise) while being engaged in other activities.	Delayed vocabulary development because of less “talk time”. Reduced focus on intellectual tasks.	Distraction of children from current activities. Reduced exposure to meaningful family interaction.	[15,20,25,26]
Media as a peacekeeper	Parents use screen devices to escape tension and concentrate on tasks such as food preparation, doing office work, or household duties.	Decreased focus and attention span. Both short and long-term speech delay	Decreased parent-child interaction. Less time for creative activities. Weakened psychological outcome.	[11,24,26]
Bedtime media use	Parents comprehending that screen time helps in calming children to sleep.	Adverse outcome on speech delivery.	Resistance to sleep Condensed sleep length	[20]
Touchscreen benefits for motor development in infants	Belief that early touch screen interaction boosts fine motor skill advancement.	Experiencing “video deficit”— difficulties while transitioning from 2D to 3D objects Troubled motor skills.	Disrupted mental flexibility. Higher Body Mass Index (BMI)	[11,14,26]
Use of smart devices as an indication of status	Societal pressure to endlessly purchase latest screen gadgets to children for status.	Delayed cognitive, and verbal advancement.	Limited outdoor play opportunities. Tendency towards a sedentary lifestyle	[2]
Underestimation of media consumption	Parents underestimating their own children’s screen viewing length.	Physical changes to brain structure Challenges in information processing.	Agitated behavioral change. Higher obesity risk Reduced sleep duration	[24]
Lack of consciousness of existing recommendations	Confusion or ignorance of existing standards, with a focus only on benefits of physical activity.	Disproportionate attainment of cognitive indicators. Possibilities of hyperactivity and inattention	Health hazards later in life Disrupted sleep and eating routine	[5]

Table 2 demonstrates that a significant number of parents allow screen time for their children due to an array of familial and societal reasons. Many parents who have newborns and younger toddlers use screen devices as a mode of distraction or a means to prevent conflict, manage household chores, or even put children to sleep. Some of them give their child the latest smart gadgets as a means of maintaining social status because of the perception of these devices as standing symbols. Some parents just view these devices as a convenient educational tool, believing screen media literacy will prevent their offspring from falling behind other children in an academic setting, while others are protective against the outside world and would choose for their children to stay indoors and be secure. Moreover, some parents underestimate their children’s screen time entirely, and others are merely oblivious to the recommendations. Such dependence on screen devices has normalized their presence in regular life, advocating to parents that screen use is an innocent and required aspect of advanced upbringing, often obscuring the potential harms associated with screen use which can be serious such as mood fluctuations, sleep difficulties, psychological and physical changes, reduced attention spans, and other health problems. Studies also highlight the influence of convincing marketing campaigns that portray screen time as absolutely imperative for educational and entertainment purposes, further concealing the challenges associated with them [28]. These findings highlight the need to promote parental awareness and mindful digital engagement during early childhood. It is therefore crucial to present parents with evidence-based tools that can balance their child’s screen use with developmental requirements.

### Strategies for Moderating Screen Time in Younger Children (RQ2)

**Figure 3.**
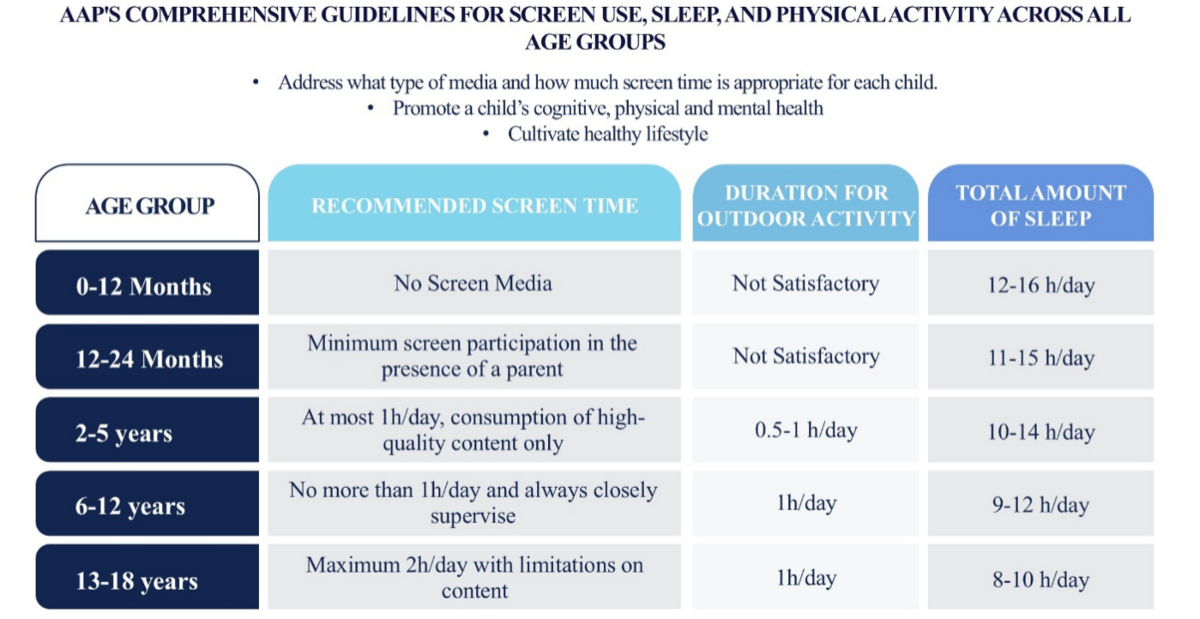
The advised total amount of screen time, physical/outside activity, and sleep for discrete age groups of children [13,17-20]. AAP: American Academy of Pediatrics.

The studies indicate that the continuous presence of smartphones and other smart gadgets within households has become an inherent component in the lives of infants and toddlers [29]. Among this significant population, roughly 72% of children aged 0‐8 years and 38% of children younger than 2 years have access to some mode of smart devices such as smartphones, iPads, or tablet devices [30,31]. Despite a plethora of studies emphasizing the short-term difficulties during infancy and continuing long-term complications in the subsequent growing phases, such smart devices remain an inevitable part of current parenting due to their ability to attract the minds of children successfully [13]. After acknowledging the consequences, authorities such as the AAP, the US Department of Health and Human Services, the Australian Department of Health and Ageing, and the Canadian Pediatric Society strongly instruct plummeting screen time as one of the significant urgent issues by entirely restraining media use on children younger than 2 years, and instigating rigorous boundaries for those between 2 and 18 years [20]. Figure 3 demonstrates the recommended duration of screen time and other developmentally supportive activities for children stated by AAP to enable healthy behaviors, such as including enough sleep, outdoor activities, and constant physical play that can successfully counterbalance screen exposure.

The results depicted that extreme screen use in a family is strongly associated with a toddler’s own screen viewing practices. Given that children younger than 2 years have very limited power over their adjacent surroundings and their actions are merely a replication of what their parents do around them, their screen habits are formed by what they perceive. For instance, studies highlight that if parents engage in more than 4 hours a day watching TV, their toddlers will be 3 times more likely to exceed the same duration [32]. Thus, parents need to regulate their own screen use and model positive examples as they are the chief contributors to their infants’ long-term well-being. Childcare providers, more specifically pediatricians, play a fundamental role in supporting well-adjusted screen habits by educating parents and continually supervising parental adherence to the guidelines during scheduled appointments, assuring their proper implementation within households [20,33]. Therefore, intervention strategies should largely entail actions taken by parents as well as childcare providers, as each of them has a unique role in the infant’s long-term betterment [33]. Given the increasing impact of modernization within home environments, the following intervention strategies demonstrated in Table 3 provide ideal directions for distinct family perspectives, and it is imperative to implement such principles as references for forthcoming actions. Considering the outcomes of various studies, 6 types of intervention approaches for parents and 3 for pediatricians have been distinguished, along with their expected outcomes as summarized in Table 3.

**Table 3.** Intervention strategies and their anticipated outcome.

Target contributor and recommended intervention strategies		Description	Expected outcome	Reference
Parents				
Restriction on child’s screen time		Consistently control screen time throughout the day, avert using any screen device during meals or sleep, and turn off devices while not in use.	Promote healthy eating behaviors. Enhance language acquisition Better quality of life	[13,15]
Limit parental screen time		Formulate home-based regulations on screen media specifically for parents.	Reduces screen time of children through modeling. Increased parent-child connectivity	[17,34]
Coviewing contents		Parents encourage co-watching high quality content, supervising what their children see and hear.	Enhances cognitive development. Encourages parent-child interaction Significantly expands vocabulary during infancy	[6,13,24]
Focus on quality over quantity		Promote educational content which uses schemes to assist language training, contains naive story structures and allows child interaction scopes.	Higher communication skills Increases prosocial behavior. Expands concentration. Encourages positive racial outlook	[6,13,15,26]
Promote child-directed and age-appropriate media content		Recommend contents for younger children that contains longer displays, provides narration hints, labeling, and structured storytelling.	Facilitates cognitive understanding. Overcomes “video deficit”. Higher school readiness	[11,17]
Promote a healthy lifestyle.		Include outdoor activity, imaginative play, a regular sleep cycle, and a nutritious diet.	Promotes locomotive skills, reduces childhood obesity, and improves metabolic health. Influence adaptive health.	[35]
Pediatricians				
Set age-appropriate “media limits”		Discuss and enforce screen media restrictions for children younger than 2 years during scheduled visits.	Fosters balanced screen behaviors at an initial stage	[20]
Encourage supervised independent play		Inform parents about independent play when they are unavailable but able to supervise their child.	Builds perseverance and problem-solving expertise while remaining under surveillance	[20,33]
Explain the value of “unplugged play”		Encourage parents to engage children in hands-on, imaginative, and performing activities that interest them.	Encourages creative thinking Expands problem solving skills.	[3]

### Developing a Holistic Strategy: A Pathway to Expand Children’s Cognitive Advancement

Studies indicate that engaged, collaborative, and informed parenting can successfully reverse the detrimental impacts of excessive screen use while fostering complete development [36]. For instance, research has confirmed that the presence of a parent who actively gives statements or explanations on the content their child watches, starting from as early as 6 months, can have a positive impact on the infant’s intellectual engagement and attention span [26]. However, modern life often restricts the scope and time for quality parent-child communications, as time-restrained parents face tight spots to implement these policies. Therefore, a balanced solution is needed that moderates the negative impacts of screen time while leveraging the advantages of using several digital tools [37]. As the research team reviewed some existing strategies to help parents balance screen time for their infants and toddlers, studies revealed some parental control mobile apps, such as Google Family Link, Apple Family Sharing, and Microsoft Family Safety, which primarily focus on screen time management for older children. However, these apps provide inadequate guidance for children younger than 3 years, which needs to be addressed through age-appropriate solutions to guarantee healthy early development.

## Discussion

### Principal Results

This study underlines that excessive screen exposure has extensive consequences on the overall well-being of infants and younger toddlers, putting this phenomenon into a different perspective. The consequences are far more serious, and they are not simply a result of screen exposure but are greatly interconnected with parental personal screen media use, attitudes, behaviors towards technology, and ignorance of the risks posed by digital devices. Parents’ reliance on screens—whether as an opportunity of defense against the outside world, a means of distraction, a perceived educational tool, or even a status symbol—plays a critical role in offering their children a smart device. It is undeniable that the presence of smart devices will further reinforce with time, augmenting the urgent necessity for appropriate and practical interventions that encourage mindful digital engagement, particularly in the case of infants and toddlers.

Major intervention strategies identified in this paper include restricting screen time for both parents and their offspring, coviewing age-appropriate programs, and prioritizing nonscreen activities as they promote healthier behavior and increase parent-child connection [13,15,17,34]. More hands-on strategies, such as quality regulation of content, are also essential as younger children often lack the decision-making capabilities of which programs to watch. Therefore, the prevailing guidelines are necessary for a constructive learning experience for the younger generation [14,26]. Pediatricians also play an essential role as part of their responsibility is to advise parents on the best actions required for their children, for instance, inspire parents to establish age-appropriate time limits and suggest both supervised and independent play according to the child’s interest. Such pursuits can cultivate a recognition of persistence and broaden their thoughts of the outside world beyond just screens [20,33].

Given the presence of technology in this modern world, it is evident that addressing the challenges related to screen time consumption requires leveraging technology itself. Studies suggest an urgent need for solutions that balance regulatory guidance with sustainable and parent-centric approaches tailored to today’s sociocultural and socioeconomic realities [38,39]. Therefore, building on the understanding of the research assembled from an examination of RQ1 and RQ2, we recommend that a mHealth app can be an empathetic, practical, and scalable tool that will make supervising children’s screen time easier for today’s busy parents. The justification for this app is plain and simple; since digital devices have become an inseparable part of our daily lives and high-tech devices are already accessible to every parent, it would be a logical and feasible decision to leverage them to tackle this issue. Additionally, it would enable parents to oversee the digital ecosystem precisely and cautiously within a household.

**Figure 4.**
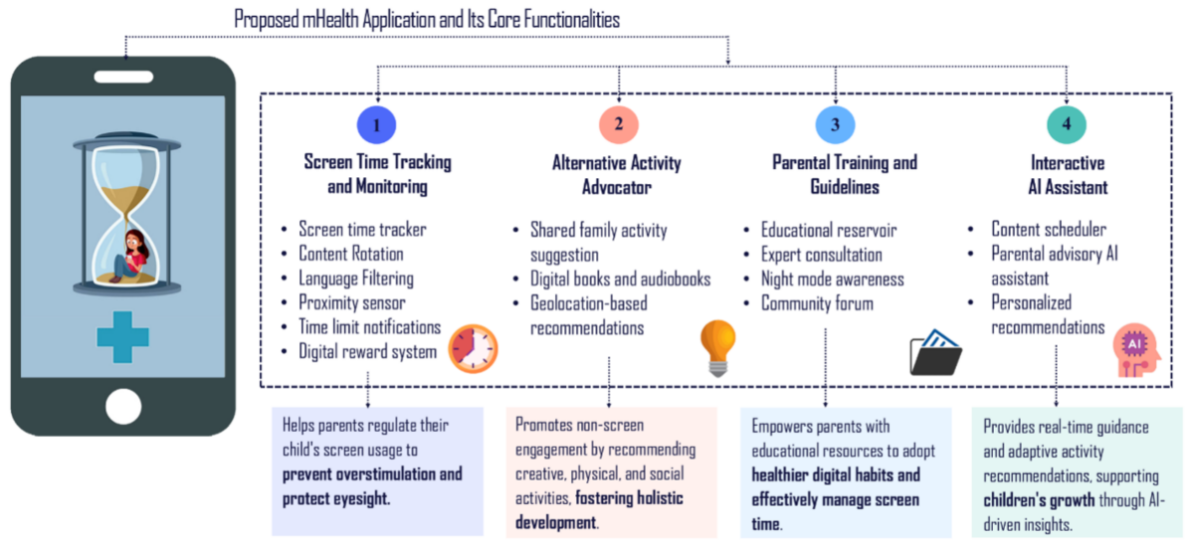
Prototype of a mHealth app along with the supervising features. AI: artificial intelligence.

The app could be built using the Scrum Agile software development methodology to ensure the progress of the app development process in an incremental and iterative way. Each sprint could drive the systematic execution of planning, design, coding, testing, and review, facilitating efficiency and high-quality outcomes. Backend development could be implemented using the Swift programming language within the Xcode IDE (Apple Inc) to facilitate development, organize events, and enhance data flow. User interface (UI) could be designed by leveraging UIKit and SwiftUI frameworks. Additionally, Swift Package Manager could be leveraged to ensure efficient dependency management, Git for version controlling of the codebase, and Firebase Software Development Kits for documentation and cross-platform app development of the scalable prototype. In delineating the direction for the development of this app, we believe four core components with their prominent features as absolutely principal for creating a holistic framework: (1) screen time tracking and monitoring (could allow parents to monitor screen use across multiple devices and set goals), (2) provision of parental training and guidelines (could provide resources to educate parents, boosting parental self-confidence in their capability for successful screen management), (3) alternative activity advocator (could propose nonscreen pursuits tailored to each child’s preferences), and finally (4) interactive artificial intelligence (AI) (could present personalized suggestions to parents, assisting them to address the challenges proactively and reducing screen dependency) (Figure 4).

### Outline of the Key Features

#### Screen Time Tracking and Monitoring

While it is not realistic for parents to completely eliminate providing their children with screen devices, they can guarantee controlled use [40]. A screen time tracking and monitoring feature can allow parents to track their toddlers’ daily screen use by showing detailed data insights across multiple devices [41]. This functionality can be accomplished by applying parental control application programming interfaces (APIs) that supervise and log screen time, along with frameworks like Xamarin or React Native, which can be used for combined codebases. To prevent cognitive fatigue and avoid overstimulation in children from repeated use of the same content, the app can periodically suggest changes in media content after a predefined duration. Younger children can struggle with multilingual content, often overwhelming their learning abilities [42-44]. Recognizing the challenges, the app could offer a language-filtering option that enables parents to streamline content into a single language, supporting early language development, supporting early language development—particularly for children with special requirements. To help parents manage screen exposure effectively, the screen time goals and alerts feature can inform parents with alerts when time limits come closer or are exceeded. Furthermore, a digital reward system can be incorporated to persuade adherence to screen time limits, applied through a gamification reward framework linked to databases to track the improvement. It is well known that prolonged screen exposure at close distances could be extremely detrimental to children’s eyesight [45]. A proximity sensor feature could use smartphone infrared sensors to alert parents if devices are held too close to infants’ and toddlers’ eyes, reducing the risk of eye strain. Both Screen Time API and the UsageStatsManager API can be operated for iOS and Android, respectively, and all the segments can be seamlessly synchronized across various devices using cloud-based services such as Firebase or AWS, confirming convenience and ease of use for parents.

#### Parental Training and Guidelines

The parental training and guideline feature could perform as an all-inclusive educational resource for parents, so that parents do not feel ignorant. The function can offer reasonable resources such as videos, articles, or infographics to delineate the outcomes of excessive screen time and suggest effective strategies for regulating it, attained using a content management system that handles the content dynamically. To make the app more reachable and user-friendly for parents with varying levels of digital literacy and access to technology, the app could incorporate a LangChain model to provide concise summaries of critical research findings as Daily Educational Push notifications, guiding them to take their next actions. This approach would guarantee that parents have easy access to an ample number of resources, removing the need for independent research and allowing them to make informed decisions regarding their child’s screen time. After clicking on the popper notification, parents could be seamlessly redirected to the detailed materials. Real-time updates can be acquired using services, for example, Contentful or Strapi. The platform can connect parents with certified child psychologists or pediatricians through audio or video calls for adapted guidance and strategies on screen time administration, using a booking system and secure calling features such as Twilio API. The blue lights emitted during screen time can significantly disrupt sleep time and sleep quality of infants and toddlers [46]. It is feasible to create collaborative tutorials using libraries such as Intro.js and connect them to the device API to design a scheduled Night mode module that offers step-by-step directions on lowering blue light emission during nighttime gadget use. Finally, a community forum module can facilitate parents in sharing tips, their experiences, hardships, and success stories on monitoring their child’s screen time so that they always feel supported in their journey. Both the front and back end of this segment can be developed by using React.js and Node.js, ensuring appropriate authentication, fundamental for this facility to operate.

#### Alternative Activity Advocator

Nonscreen activities play a vital role in the holistic development of children aged 0‐3 years, as this period determines future behavioral and health outcomes [47]. While outdoor play promotes physical health and improves motor skills, shared family activities and social interactions improve communication skills [48]. It can be challenging for parents to come up with ideas to lure their kids away from screens. The alternative activity advocator feature can be instrumental in promoting nonscreen engagement for children. Subsections falling within this section can incorporate (1) Shared Family Activities, which could offer constructive, age-appropriate family activities. Existing parental control techniques, such as Google Family Link, Apple Family Sharing, and Microsoft Family Safety, emphasize screen time tracking but do not offer any guidance for alternative activities. The proposed feature could include diverse activity categories based on a child’s creative, active, social, and educational interests and recommend tasks accordingly. For instance, interactive activities like listening to nursery rhymes to nurture creativity, baby yoga to encourage physical activity, or a scavenger hunt to build an adventurous mindset [49,50]. Such activities can significantly help children meet milestones, establish a foundation for parent-child bonding, and build their motor, sensory, and communication skills. (2) The next subfeature could suggest the Provision of Digital and Audiobooks, delivering an assorted library of books suitable for a child’s age and intensity of reading. The level of engagement will be higher if the picture books include colorful visuals, simple language, and interactive storytelling options to capture the attention of children, particularly infants or younger toddlers [51]. (3) Finally, the app could further incorporate Geolocation-Based Recommendations to identify nearby parks, playgrounds, or family activity centers where parents can arrange playdates with other families [52]. This way, parents can discover safe and engaging environments for their children to socialize with same-age children and develop their social interaction skills at an early age. A decision tree algorithm could be used, driven by data collected through in-app surveys and stored in a NoSQL database like MongoDB for flexibility to attain such distinctive offerings. Activity recommendation algorithms can be used using Python’s scikit-learn or comparable libraries. These features would reduce reliance on screens while fostering creativity, physical activity, and real-world interaction in an organized manner.

#### Interactive AI

The mHealth app can incorporate another innovative attribute by channeling the power of AI to present parents with adapted guidance. An AI-based Content Scheduler can be developed to offer busy parents flexible options for planning their child’s activities during mealtimes, play hours, and bedtime, offering real-time support. Automatic AI scheduling could leverage collaborative filtering, content-based recommendations, and context awareness to generate developmentally appropriate schedules. A Parental Advisory AI assistant can be established, using natural language processing libraries such as NLTK or spaCy in Python, incorporated with an AI chatbot framework, for instance, Rasa or Microsoft Bot Framework. Additionally, an AI-assisted Personalized Activity Advocator, leveraging the LangChain framework and advanced large language model capabilities, could deliver tailored recommendations for nonscreen activities and developmentally appropriate digital educational content [53-55]. Drawing from user survey data, collaboration history, and professional input, this feature could support the fundamental cognitive development goals for infants and toddlers. Additionally, A/B testing could be used to continuously improve the algorithms based on user feedback, ensuring relevance and accuracy.

To ensure strategic scalability, the app could primarily launch a freemium model, proposing a basic version tailored for home-based ecosystem to allow widespread adoption. Simultaneously, a premium model, available through subscription or payment, could be introduced for daycare or childcare environments, generating sustainable revenue. The basic version can be upgraded to a premium version with a subscription at any time, ensuring accessibility and long-term market success. We believe that by integrating this innovative solution along with all the recommended features, the app will promote healthy cognitive and emotional development, establish positive media habits, and adapt seamlessly to busy family dynamics.

All in all, by leveraging the revolutionary principles of information technology, machine learning, and AI, the proposed mHealth app could become a one-stop solution for confronting the challenges modern parents face in managing screen time for their children younger than 3 years. This novel app can act as an exclusive knowledge hub for screen time management, parenting guidelines, tips, and expert advice. A comprehensive framework, easy-to-understand UI, and integration of advanced functionalities are future ways to maintain the digital lifestyles of toddlers and infants.

### Limitations

The outcomes of this research contribute to a deeper understanding of the significant correlation between screen time consumption, parental behaviors, and early childhood development. However, it is compulsory to address specific limitations. Existing studies often fail to consider individual variability among children and different family dynamics, such as socioeconomic factors, parenting styles, and cultural influences. Consequently, this oversight restricts the relevance or applicability of findings across discrete socioeconomic populations. Our research concentrated on general patterns about the effects of screen time without considering different family structures, but recognizes the necessity for future research to further examine these nuances.

The complex and continuously evolving character of the digital landscape presents difficulties in effectively managing screen time. Therefore, the proposed mHealth app, including the existing guidelines, must be pliable and capable of addressing emerging challenges presented to it. Leveraging AI and machine learning to offer continuous updates would provide parents with more control over the watched contents, guaranteeing continual efficacy.

Ensuring user engagement is another challenging procedure. Integrating AI with diverse user input data may demand model training data adjustments. Future studies could use iterative refinement cycles and investigate additional algorithms to ensure optimal performance. Furthermore, the usability evaluation may reveal unexpected UI challenges. Executing iterative usability testing cycles with small groups could enable mitigating this challenge, allowing for design refinement.

### Conclusions

A significant proportion of children begin engaging with smartphones and other screen devices at an earlier stage and for a longer period than childcare authorities advocate. The clear effects of the digital landscape and smart device availability highlighted in this study uncover critical research questions about program content, parental behavior, and the possible direct effects on infants and toddlers; highlighting that there is a pressing need for holistic, evidence-based approaches to address the developmental challenges associated with excessive screen time consumption among children aged 0‐3 years. In response to this issue, the paper proposes integrating technology into the solution through a mHealth app constructed to balance the developing requirements of children with the pressures of modern parenting. The novel solution, along with its innovative features, could become a valuable partner in helping parents face repercussions, offering massive support from early childhood. The beneficiaries of this solution are the children themselves, who would obtain immense enrichment in their overall cognitive and emotional advancement through reasonable and practical screen use. The findings should also inspire parents and pediatricians to discuss children’s screen habits, warn researchers to unpack the contradictions in the existing literature, and discover potential prospects for defensive interventions. Finally, the paper underlines the importance of further research so that the solutions can appear to be readily applicable and adaptable across all naturalistic home-based settings. With strong potential for commercialization and industry impact, the proposed mHealth app is poised to transform child well-being and promote lasting benefits for families and communities.

## Supplementary material

10.2196/60355 Checklist 1 PRISMA checklist.

## Abbreviations

AAP: American Academy of Pediatrics
AI: artificial intelligence
API: application programming interface
PRISMA: Preferred Reporting Items for Systematic Reviews and Meta-Analyses
UI: user interface

## References

[1] Kaur N, Gupta M, Malhi P, Grover S (2021). A multicomponent intervention to reduce screen time among children aged 2-5 years in Chandigarh, North India: protocol for a randomized controlled trial. JMIR Res Protoc.

[2] Lauricella AR, Wartella E, Rideout VJ (2015). Young children’s screen time: the complex role of parent and child factors. J Appl Dev Psychol.

[3] Linebarger DL, Vaala SE (2010). Screen media and language development in infants and toddlers: an ecological perspective. Dev Rev.

[4] Jago R, Fox KR, Page AS, Brockman R, Thompson JL (2010). Parent and child physical activity and sedentary time: do active parents foster active children?. BMC Public Health.

[5] Domingues‐Montanari S (2017). Clinical and psychological effects of excessive screen time on children. J Paediatrics Child Health.

[6] Kaur N, Gupta M, Malhi P, Grover S (2019). Screen time in under-five children. Indian Pediatr.

[7] Anjum N, Hasan M, Salma SU, Zhao L, de Clemente MV, Sakib N (2025). ScreenSafeFuture: a parent-empathetic and pragmatic mhealth application for toddlers’ brain development addressing screen-addiction challenges. SoftwareX.

[8] Anjum N, Hasan M, Karim E, Ahamed SI, Garefino A, Sakib N (2024). Unpacking early digital addiction and developmental challenges in young children: a scoping review towards rethinking digital habits (preprint). J Med Internet Res.

[9] Brauchli V, Sticca F, Edelsbrunner P, von Wyl A, Lannen P (2024). Are screen media the new pacifiers? the role of parenting stress and parental attitudes for children’s screen time in early childhood. Comput Human Behav.

[10] Xu H, Wen LM, Rissel C (2015). Associations of parental influences with physical activity and screen time among young children: a systematic review. J Obes.

[11] Hetherington E, McDonald S, Racine N, Tough S (2020). Longitudinal predictors of self-regulation at school entry: findings from the all our families cohort. Children (Basel).

[12] Whiting S, Buoncristiano M, Gelius P (2021). Physical Activity, Screen Time, and Sleep Duration of Children Aged 6-9 Years in 25 Countries: An Analysis within the WHO European Childhood Obesity Surveillance Initiative (COSI) 2015-2017. Obes Facts.

[13] Tandon PS, Zhou C, Lozano P, Christakis DA (2011). Preschoolers’ total daily screen time at home and by type of child care. J Pediatr.

[14] Foreman J, Salim AT, Praveen A (2021). Association between digital smart device use and myopia: a systematic review and meta-analysis. Lancet Digit Health.

[15] Byeon H, Hong S (2015). Relationship between television viewing and language delay in toddlers: evidence from a Korea national cross-sectional survey. PLoS One.

[16] Zimmerman FJ, Christakis DA (2007). Associations between content types of early media exposure and subsequent attentional problems. Pediatrics.

[17] Panjeti-Madan VN, Ranganathan P (2023). Impact of screen time on children’s development: cognitive, language, physical, and social and emotional domains. MTI.

[18] Pagani LS, Fitzpatrick C, Barnett TA (2013). Early childhood television viewing and kindergarten entry readiness. Pediatr Res.

[19] Madigan S, Browne D, Racine N, Mori C, Tough S (2019). Association between screen time and children’s performance on a developmental screening test. JAMA Pediatr.

[20] Brown A, Council on Communications and Media (2011). Media use by children younger than 2 years. Pediatrics.

[21] Wilkinson C, Low DF, Gluckman SP Screen time: the effects on children’s emotional, social, and cognitive development. Koi Tū Centre for Informed Futures.

[22] Weirauch V, Soehnchen C, Burmann A, Meister S (2024). Methods, indicators, and end-user involvement in the evaluation of digital health interventions for the public: scoping review. J Med Internet Res.

[23] Arksey H, O’Malley L (2005). Scoping studies: towards a methodological framework. Int J Soc Res Methodol.

[24] Zimmerman FJ, Christakis DA, Meltzoff AN (2007). Television and DVD/video viewing in children younger than 2 years. Arch Pediatr Adolesc Med.

[25] Duch H, Fisher EM, Ensari I, Harrington A (2013). Screen time use in children under 3 years old: a systematic review of correlates. Int J Behav Nutr Phys Act.

[26] Gingold JA, Simon AE, Schoendorf KC (2014). Excess screen time in US children: association with family rules and alternative activities. Clin Pediatr (Phila).

[27] Certain LK, Kahn RS (2002). Prevalence, correlates, and trajectory of television viewing among infants and toddlers. Pediatrics.

[28] Lammers SM, Woods RJ, Brotherson SE, Deal JE, Platt CA (2022). Explaining adherence to American academy of pediatrics screen time recommendations with caregiver awareness and parental motivation factors: mixed methods study. JMIR Pediatr Parent.

[29] Auxier B, Anderson M, Perrin A, Turner E (2020). Parenting children in the age of screens. Pew Research Center.

[30] Harrison E, McTavish M (2018). ‘i’Babies: infants’ and toddlers’ emergent language and literacy in a digital culture of iDevices. Journal of Early Childhood Literacy.

[31] Lillard AS, Peterson J (2011). The immediate impact of different types of television on young children’s executive function. Pediatrics.

[32] Visier-Alfonso ME, Sánchez-López M, Rodríguez-Martín B (2023). Parents’ perceptions of children’s and adolescents’ use of electronic devices to promote physical activity: systematic review of qualitative evidence. JMIR Mhealth Uhealth.

[33] Guellai B, Somogyi E, Esseily R, Chopin A (2022). Effects of screen exposure on young children’s cognitive development: a review. Front Psychol.

[34] Papadakis S, Zaranis N, Kalogiannakis M (2019). Parental involvement and attitudes towards young Greek children’s mobile usage. Int J Child Comput Interact.

[35] Guram S, Heinz P (2018). Media use in children: American academy of pediatrics recommendations 2016. Arch Dis Child Educ Pract Ed.

[36] Ponti M, Bélanger S, Grimes R (2017). Screen time and young children: promoting health and development in a digital world. Paediatr Child Health.

[37] Ponti M (2023). Screen time and preschool children: promoting health and development in a digital world. Paediatr Child Health.

[38] Schmidt ME, Haines J, O’Brien A (2012). Systematic review of effective strategies for reducing screen time among young children. Obesity (Silver Spring).

[39] Alkalash SH, Alshamrani FA, Alharthi SA (2023). Parents’ knowledge on, attitude toward, and practice of screen time exposure regulation of their children under six years of age in Western Region, Saudi Arabia. Cureus.

[40] Hiniker A, Suh H, Cao S, Kientz JA (2016). Screen time tantrums: how families manage screen media experiences for toddlers and preschoolers.

[41] Gentile DA, Reimer RA, Nathanson AI, Walsh DA, Eisenmann JC (2014). Protective effects of parental monitoring of children’s media use: a prospective study. JAMA Pediatr.

[42] Dewaele JM, van Oudenhoven JP (2009). The effect of multilingualism/multiculturalism on personality: no gain without pain for third culture kids?. Int J Multiling.

[43] Dumetz J, Vichnyakova A (2021). Unexpected disadvantages of a simultaneous quadrilingual upbringing, a case study citation. Int J Teach Educ.

[44] Ren W (2024). The difficulties and challenges of learning English in a multilingual environment. Lect Notes Lang Lit.

[45] Priftis N, Panagiotakos D (2023). Screen time and its health consequences in children and adolescents. Children (Basel).

[46] Emond JA, O’Malley AJ, Neelon B, Kravitz RM, Ostbye T, Benjamin-Neelon SE (2021). Associations between daily screen time and sleep in a racially and socioeconomically diverse sample of US infants: a prospective cohort study. BMJ Open.

[47] Craig D, Trina NA, Monsur M (2024). Effective nature-based outdoor play and learning environments for below-3 children: a literature-based summary. Int J Environ Res Public Health.

[48] Sugiyama M, Tsuchiya KJ, Okubo Y (2023). Outdoor play as a mitigating factor in the association between screen time for young children and neurodevelopmental outcomes. JAMA Pediatr.

[49] Activities for baby using everyday household items. Pathways.

[50] Markham D Your age-by-age guide to screen-free activities your child can do with minimal supervision. Peaceful Parent Happy Kids.

[51] Lawless Z How engaged are infants and toddlers while picture book?. Undergraduate Honors Theses.

[52] Putnick DL, Trinh MH, Sundaram R (2023). Displacement of peer play by screen time: associations with toddler development. Pediatr Res.

[53] Mattern D, Lopez FM, Ernst MR, Aubret A, Triesch J (2022). MIMo: a multi-modal infant model for studying cognitive development in humans and ais.

[54] Sung IY, Jeon JY, Yun KJ (2020). Development of tablet personal computer-based cognitive training programs for children with developmental disabilities whose cognitive age is less than 4 years. Medicine (Baltimore).

[55] Lu P, Feng X (2024). Personalized recommendation algorithm in AI-assisted learning system.

