# Supplementary material for: Early Digital Engagement Among Younger Children and the Transformation of Parenting in the Digital Age From an mHealth Perspective: Scoping Review
Source: JMIR Pediatr Parent. 2025 Jul 3;8:e60355. doi: 10.2196/60355 (PMC12244279; doi:10.2196/60355)
Supplement: Checklist 1 [file pediatrics-v8-e60355-s001.docx]

| **Page 1Section and Topic** | **Item #** | **Checklist item** | **Location where item is reported** |
| --- | --- | --- | --- |
| **TITLE** | | |  |
| Title | 1 | Transforming Parenting in Digital Age: A Scoping Review through an mHealth Perspective on Early Digital Engagement in Younger Children | Page 1 |
| **ABSTRACT** | | |  |
| Abstract | 2 | The abstract provides a structured summary that includes the purpose of the scoping review, a brief description of the methods (databases searched, PRISMA-SCR adherence, and data synthesis processes), key results such as identified themes and intervention strategies, and conclusions emphasizing the implications of the findings. The abstract aligns with scoping review reporting norms, detailing the focus on parental behavior and mHealth solutions. | Page 1 |
| **INTRODUCTION** | | |  |
| Rationale | 3 | The increasing inclination of excessive screen time amongst children has become a common problem recently, with evidence pointing to severe impacts on critical aspects of children's early childhood development including lower academic achievements, increased anxiety, difficulties in learning languages, and other developmental setbacks. With competing responsibilities and limited scope of supervision, screen devices have appeared as a convenient childcare alternative and a stress management tool, serving as a helping hand for struggling parents, leading to nearly 70% of parents disregarding the AAP recommendations. These observations highlight a disconnect between existing guidelines for limiting screen time in infants. For these reasons, a scoping review through an mHealth viewpoint was conducted in order to systematically map the research done in this area, as well as to identify any existing gaps in knowledge | Page 2 |
| Objectives | 4 | To address the challenges, the paper addresses the following three research questions:  **RQ1:** How does the lack of knowledge about the potential risks of excessive screen time on a child’s cognitive development influence the parental decision-making process?  **RQ2:** What alternative interventions have been identified that parents can utilize to successfully distract children from having excessive screen time and support their intellectual capabilities in a home-based setting?  **RQ3:** How can these alternate solutions be effectively incorporated into a holistic approach that alleviates the negative effects of extreme screen exposure on young children's overall advancement? | Page 2 |
| **METHODS** | | |  |
| Eligibility criteria | 5 | Publications were eligible for inclusion if they were written in English, published in interdisciplinary conference proceedings, respectable journals, or as strategy reports constructed by a government organization. Broad eligibility criteria were used to maximize the possibility of finding appropriate information, such as the studies demonstrated expertise in child health science, child psychology, explored alternative strategies to reduce screen dependency such as outdoor activity or mHealth applications and deeply engaged in a discussion concerning the detection of impacts, correlations, and solutions for children aged 36 months and younger. Researchers attended an exclusion criteria to ensure consistency in screening and to maximize the possibility of excluding unsatisfactory literature. The exclusion process involved two stages of screening, an initial removal of duplicate and irrelevant articles based on titles and abstracts followed by an exclusion of studies that were not peer-reviewed, published in grey literature and unrelated especially to child cognitive or behavioral growth. | Page 4 |
| Information sources | 6 | The literature review comprised journal papers, peer-reviewed articles, and official reports which were sourced and finalized from academic databases such as Google Scholar, JMIR, PubMed, IEEE Xplore, and Elsevier. Advanced search features, such as MeSH terms in PubMed, along with targeted searches including Pediatrics and Parenting, mHealth and uHealth within JMIR were used to recognize the role of child- and parent-centered approaches using mobile applications, information technologies, engineering solutions and expand the scope of infants and toddlers’ health outcomes. | Page 4 |
| Search strategy | 7 | Literature Search Performed: March 16, 2024  Google Scholar  JMIR: Pediatrics and Parenting, mHealth and uHealth  PubMed: MeSH  IEEE Xplore  Elsevier  Google Play Store  Apple App Store | Page 4 |
| Selection process | 8 | To increase consistency among reviewers, all reviewers screened the same 93 publications, discussed the results and amended the screening and data extraction manual before beginning screening for this review. Reviewers sequentially evaluated the titles, abstracts and then full text of all publications identified by our searches for potentially relevant publications. |  |
| Data collection process | 9 | All reviewers individually collected data from each report, and collected data from study investigations that significantly contributes to our research questions. |  |
| Data items | 10a | We abstracted data on the characteristics of the included studies (e.g., author, year of publication, country of origin), study design (e.g., qualitative, quantitative, mixed methods), participant demographics (e.g., age group, socioeconomic background, parental involvement), and research focus (e.g., cognitive development, screen media exposure, interventions). Specific details of screen media use (e.g., average daily screen time, type of content, and parental attitudes) and intervention strategies (e.g., co-viewing, use of mHealth applications, alternative activities) were also extracted. Additionally, we documented contextual factors (e.g., regional screen media trends, technological accessibility) and reported outcomes, including developmental challenges, cognitive growth, and parental engagement. |  |
|  | 10b | Data from included studies were systematically extracted and organized into a pre-developed data charting form using Excel. The form included fields for bibliographic details, study characteristics, participant demographics, study outcomes, and recommendations. The charting form was iteratively refined based on team discussions and pilot extractions from a subset of studies to ensure consistency and relevance. Each included study was independently reviewed by two researchers, and discrepancies were resolved through discussion with a third reviewer to ensure accuracy and comprehensiveness. |  |
| Study risk of bias assessment | 11 | All reviewers individually collected data from each report, and collected data from study investigations that significantly contributes to our research questions. |  |
| Effect measures | 12 | This scoping review employed a qualitative synthesis approach, with no quantitative effect measures such as risk ratios or mean differences calculated. The outcomes were narratively described, emphasizing key themes and findings from the included studies. | Page 5-9 |
| Synthesis methods | 13a | Publications were eligible for inclusion if they were written in English, published in interdisciplinary conference proceedings, respectable journals, or as strategy reports constructed by a government organization. Broad eligibility criteria were used to maximize the possibility of finding appropriate information, such as the studies demonstrated expertise in child health science, child psychology, explored alternative strategies to reduce screen dependency such as outdoor activity or mHealth applications and deeply engaged in a discussion concerning the detection of impacts, correlations, and solutions for children aged 36 months and younger. | Page 4 |
|  | 13b | Missing or incomplete data from individual studies were noted but not imputed or converted. Only data explicitly reported in the included studies were used in the synthesis to maintain accuracy and transparency. | Page 5 |
|  | 13c | Results were synthesized and visually presented through tables (e.g., intervention strategies and their expected outcomes in Table 2) and thematic summaries. | Page 6, 8 |
|  | 13d | A narrative synthesis was used to group results into themes such as parental attitudes, intervention strategies, and developmental outcomes. The choice of narrative synthesis was due to the qualitative nature of the studies and the absence of quantitative data. | Page 5 |
|  | 13e | N/A |  |
|  | 13f | N/A |  |
| Reporting bias assessment | 14 | Risk of bias was not formally assessed, as the review focused on a qualitative synthesis. |  |
| Certainty assessment | 15 | Confidence in the findings was strengthened by including peer-reviewed studies and reports from reputable sources. |  |
| **RESULTS** | | |  |
| Study selection | 16a | The results of the search and selection process are detailed in the PRISMA flowchart (Figure 2). 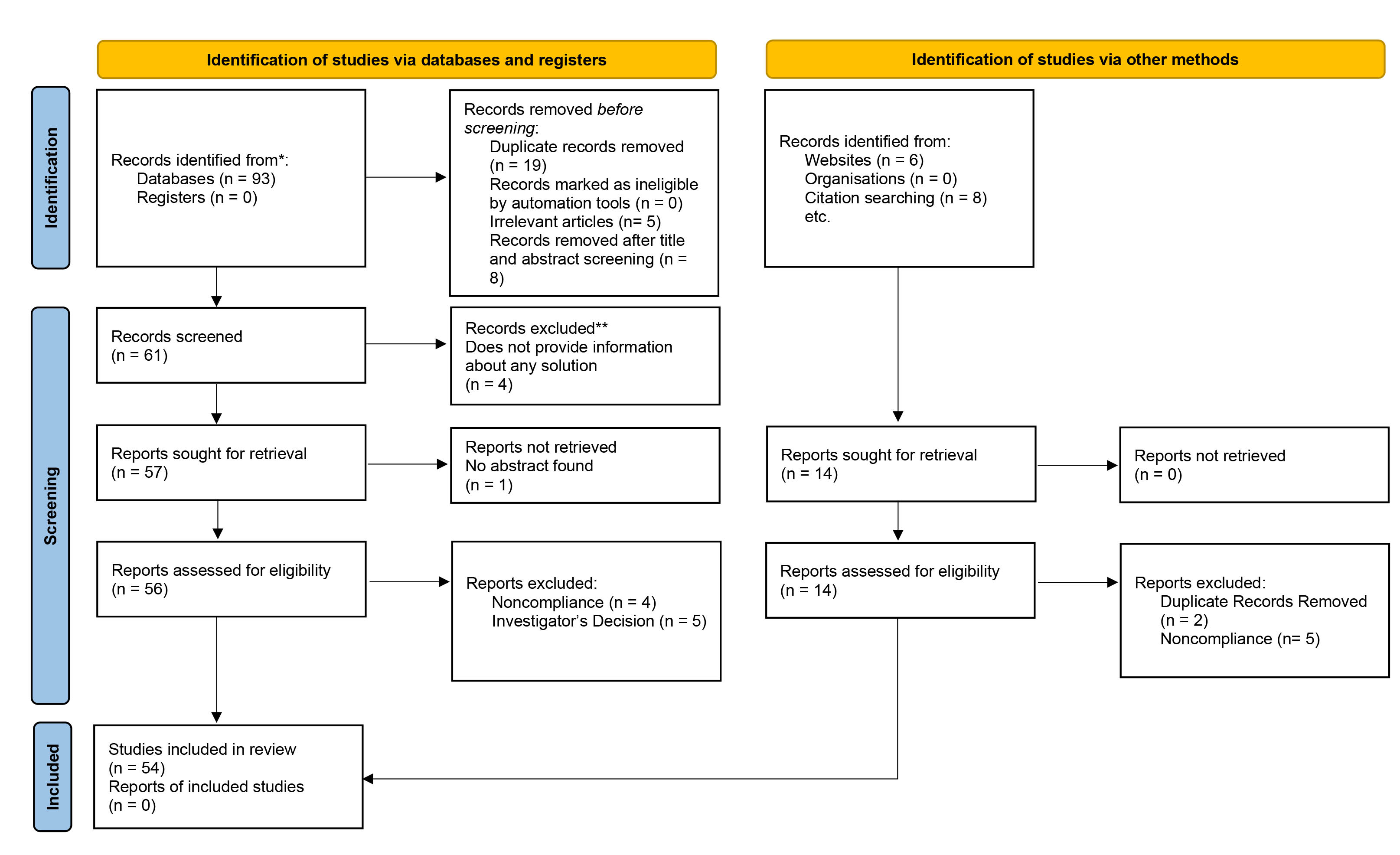 | Page 5 |
|  | 16b | Studies were excluded primarily due to irrelevance to the research questions and methodological inadequacies. Specific exclusions are detailed in Figure 2. | Page 5 |
| Study characteristics | 17 | Included studies are cited throughout the results section. Their characteristics include study design (qualitative, quantitative), population (children aged 0-36 months), and focus (screen time impacts, parental behaviors, intervention strategies). | Page 5-9 |
| Risk of bias in studies | 18 | N/A |  |
| Results of individual studies | 19 | Individual study results are summarized narratively and in structured tables (e.g., Tables 1 and 2), presenting outcomes such as cognitive, behavioral, and physical impacts of screen time. | Page 6, 8 |
| Results of syntheses | 20a | Syntheses were conducted narratively by grouping studies into themes such as parental attitudes, screen time impacts, and interventions. | Page 5-9 |
|  | 20b | Results are presented narratively and in tabular form to emphasize thematic findings. | Page 6, 8 |
|  | 20c | Possible causes of heterogeneity, such as differences in parental attitudes, cultural contexts, and socio-economic factors, are discussed narratively. Subgroup analyses were not conducted due to the descriptive nature of the review. | Page 5-9 |
|  | 20d | N/A |  |
| Reporting biases | 21 | Reporting biases were not formally assessed, but the potential for bias due to the exclusion of non-English studies and grey literature is acknowledged. | Page 4 |
| Certainty of evidence | 22 | Certainty of evidence was not formally assessed using GRADE or similar tools. |  |
| **DISCUSSION** | | |  |
| Discussion | 23a | The findings emphasize the developmental challenges posed by excessive screen use and align with existing evidence highlighting its cognitive, behavioral, and social impacts. They validate prior recommendations for parental education and interventions. | Page 9-13 |
|  | 23b | The review acknowledges that existing studies often overlook variability among children and family dynamics, such as socioeconomic status and cultural influences. This limits the generalizability of findings across diverse populations. | Page 13 |
|  | 23c | Limitations include the exclusion of non-English studies and grey literature, which may have excluded valuable insights. The review relied on qualitative synthesis, which does not provide statistical precision. | Page 13 |
|  | 23d | The results advocate for the development of mHealth applications as practical tools to balance technology use in families. Policymakers and healthcare professionals should focus on supporting parents in implementing effective screen management practices. | Page 9-13 |
| **OTHER INFORMATION** | | |  |
| Registration and protocol | 24a | This review was not registered in a formal review registry. |  |
|  | 24b | N/A |  |
|  | 24c | N/A |  |
| Support | 25 | N/A |  |
| Competing interests | 26 | N/A |  |
| Availability of data, code and other materials | 27 | Data extraction templates and other review materials are not publicly available but can be provided upon request. |  |

*From:*  Page MJ, McKenzie JE, Bossuyt PM, Boutron I, Hoffmann TC, Mulrow CD, et al. The PRISMA 2020 statement: an updated guideline for reporting systematic reviews. BMJ 2021;372:n71. doi: 10.1136/bmj.n71. This work is licensed under CC BY 4.0. To view a copy of this license, visit <https://creativecommons.org/licenses/by/4.0/>
